# Emergence of unique recombinant forms (URFs) in Indian HIV-1 epidemic: data from nationwide clinical cohort between 2007 and 2011

**DOI:** 10.1186/1742-4690-9-S2-P151

**Published:** 2012-09-13

**Authors:** U Neogi, S Gupta, A Shet, A De Costa, RL Laishram, A Wanchu, V Diwan, AC Banerjea, A Sonnerborg

**Affiliations:** 1 St. John’s Medical College, Bangalore, India; 2 Department of Pediatrics, St. John's Medical College Hospital, Bangalore, India; 3 Division of Global Health, Karolinska Institutet, Stockholm, Sweden; 4 Regional Institute of Medical Science, Imphal, India; 5 Department of Internal Medicine, PGIMER, Chandigarh, India; 6 Department of Public Health and Environment, R.D. Gardi Medical Colleg, Ujjain, India; 7 National Institute of Immunology, New Delhi, India; 8 Divisions of Infectious Diseases, Department of Medicine Huddinge, Karolinska Institutet, Sweden

## Background

Current epidemiological studies in India have been limited to single or localized geographic settings within the country. In this study we aim to cherecterised the nationwide distribution pattern of the HIV-1 subtypes based on the data collected from clinical cohorts from 7 provinces from four regions in India (northern, north-eastern, central and southern).

## Methods

Blood samples were collected from 212 HIV-1 seropositive subjects between 2007 and 2011. HIV-1 subtypes were determined using at least two or three viral genes, gag, pol, and env using maximum likelihood tree. Recombination events were identified using RIP ver 3 tools followed by breakpoints analysis in Simplot version 3.5.1 and fragment-specific phylogenetic analysis.

## Results

When a single gene was used for subtype determination, the mean proportion of HIV-1C, B and A1 were 95.9%, 1.9% and 0.2% respectively while recombinants constituted 2.1%. The overall prevalence of URFs (BC/A1C) increased significantly to 10% when two (p<0.01) or three genes (p=0.02) were used. All the A1 and B strains, identified in the single-gene approach, were re-identified as URFs. Detailed analysis indicated that the B segment of the URF_BC probably originated from China/Thailand, while the A1 segments originated from eastern Africa. Among the four geographical regions examined, a high proportion of the recombinant strains based on the two-gene approach were identified in north-eastern (46.7%) and northern (18.5%) followed by southern India (5%), while central India appeared to have a concentrated subtype C epidemic (100%).

## Conclusion

The rapid and continuous evolution of the HIV-1 epidemic in India was evidenced by the increase of recombinant strains. The use of multiple genes, rather than a single gene to identify HIV-1 subtypes can reduce the chances of false identification. These results warrant an urgent need for continued molecular surveillance to guide efficient disease intervention strategies and develop an effective subunit-based vaccine.

